# Conventional Radiology in Deep Seated Facial Hemangioma: A Case Report

**DOI:** 10.7759/cureus.35186

**Published:** 2023-02-19

**Authors:** Simran D Badki, Vidya Lohe, Rahul Bhowate, Ravindra P Kadu, Ravikant Sune, Mayur B Wanjari

**Affiliations:** 1 Department of Oral Medicine and Radiology, Sharad Pawar Dental College and Hospital, Datta Meghe Institute of Higher Education and Research, Wardha, IND; 2 Department of Pathology, Jawaharlal Nehru Medical College, Datta Meghe Institute of Higher Education and Research, Wardha, IND; 3 Department of Research and Development, Jawaharlal Nehru Medical College, Datta Meghe Institute of Higher Education and Research, Wardha, IND

**Keywords:** intramuscular, phleboliths, muscles, calcifications, hemangioma

## Abstract

Hemangioma is congenital or traumatic in origin, and it is caused due to atypical build-up of the blood vessel. It is a painless benign condition with typical characteristic clinical features. These generally occur in the first three decades of life with no gender predispositions. A plain soft tissue radiograph can demonstrate phleboliths and aid in diagnosing an intramuscular hemangioma. The present report is a rare deep-seated facial hemangioma involving various facial muscles with multiple phleboliths; characteristics clinical and radiological features. A 22-year-old male patient reported a complaint of swelling on the right side of the jaw. Conventional radiography showed the right cheek’s soft tissue and multiple round, target-like radiopacities of variable sizes.

## Introduction

The word "hemangioma" originates from Greek literature where haema is "blood"; angeio means "vessel," and oma is "tumour." The hemangioma can be congenital or traumatic in origin and is an atypical build-up of blood vessels in the skin or internal organs. It is also known as vascular nevus containing unorganized blood vessels connected to the central vein. Intramuscular hemangioma (IMH) has about 1% of cases and is typically seen in the trunk or limb's skeletal muscles [1]. In the head and neck, only 13.8% of IMHs are found, the masseter muscle being the most common site [2]. Only 8% of cases can be diagnosed pre-operatively because these tumours are rare due to the overlying parotid gland and their deep intramuscular location [2,3].

Initial diagnosis is commonly considered a parotid tumour due to the location, whereas hemangiomas are barely suspected [4]. The patient usually complains of a slowly growing and often painless mass. The mass is often compressible, with pulsations and thrills with no gender predilection [4,5]. The calcifications and formation of phlebolith are characteristic features of these hemangiomas [5]. The following case report of deep-seated facial hemangioma presents the clinical and diagnostic paraclinical workup and treatment choices.

## Case presentation

**Figure 1:**
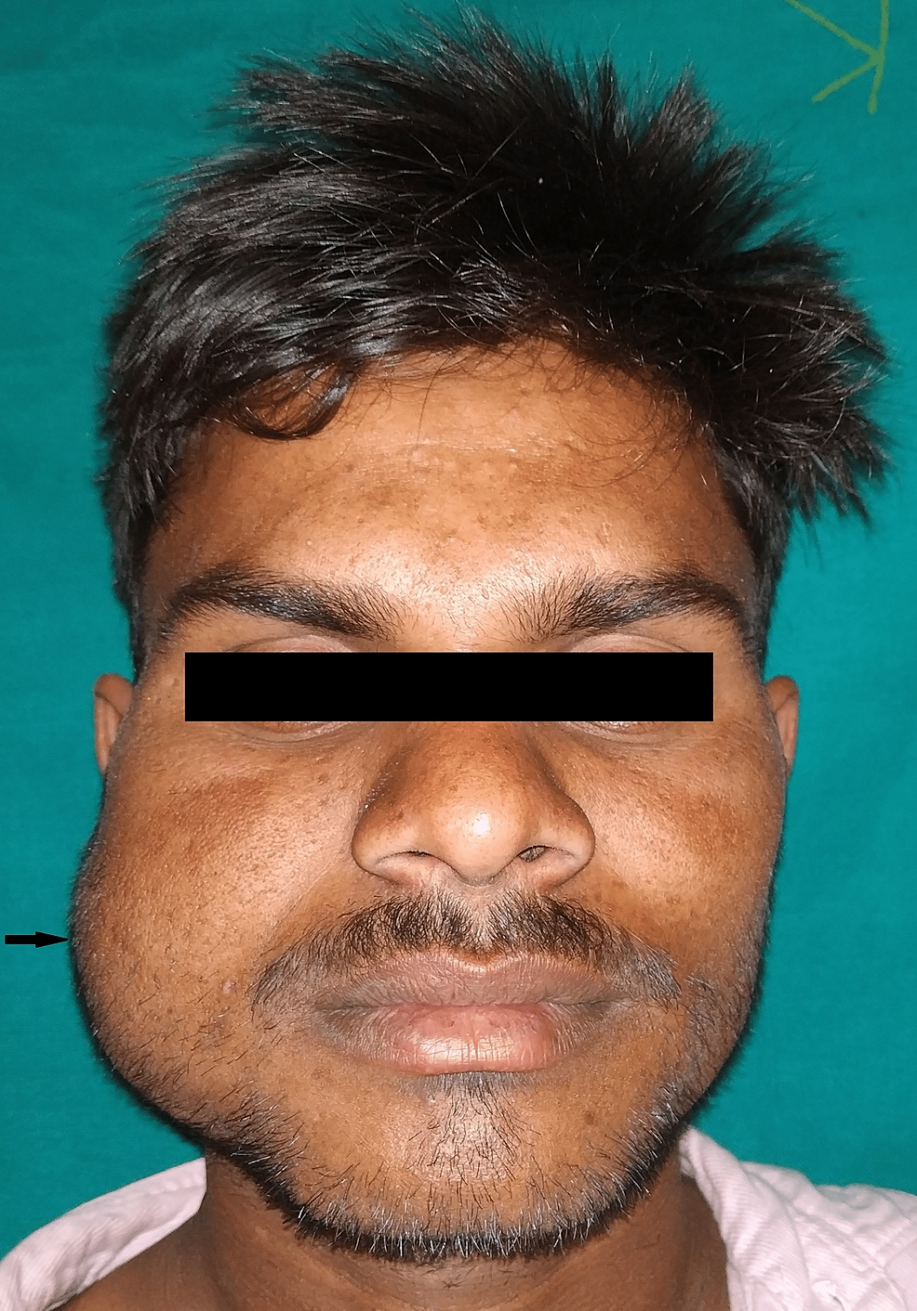
Swelling present on the right side of the face The swelling extends from the corner of the mouth to the tragus of the ear.

A 22-year-old male patient reported swelling without pain on the right side of his face since childhood as the chief complaint. On further interrogation, the patient said that the swelling had been growing gradually over the years and has now become of the present size, which was approximately fist-sized. The patient did not recall any history of trauma. The extraoral examination demonstrated a swelling on the face's right side, extending supero-inferiorly from the right eye's outer canthus to the mandible's lower border and anteroposteriorly from the right corner of the mouth to the pre-tragus region (Figure 1).

**Figure 2:**
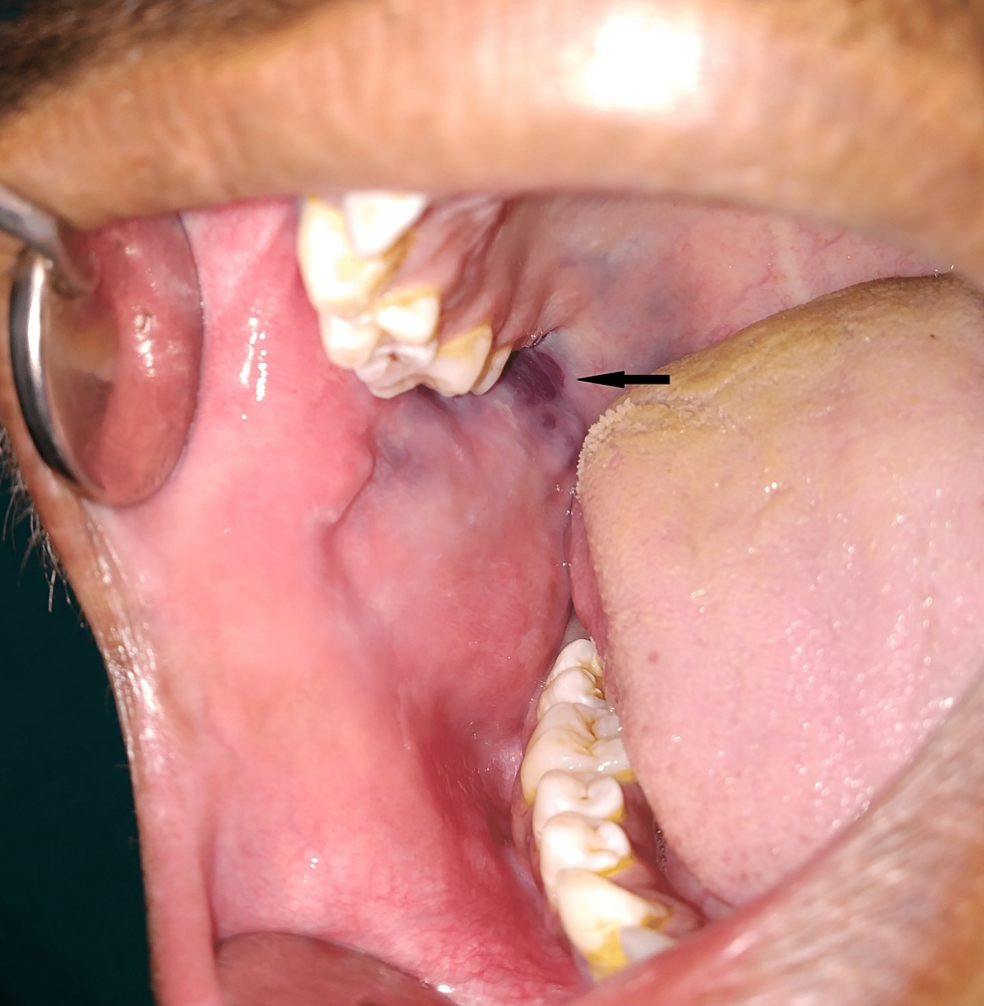
Intra-oral image The arrow is showing bluish-red discoloration in the posterior part of the right side buccal mucosa extending up to the right side soft palatal region.

The painless swelling was fluctuant, compressible, and soft in consistency on palpation. There was a slight rise in temperature over the surface of the swelling. On temporomandibular joint examination, the deviation was present towards the left side while closing the jaw without any pain, crepitus, or clicking sound. Intra-oral examination revealed bluish-red discolouration in the posterior part of the right side buccal mucosa extending up to the right side soft palatal region (Figure 2).

**Figure 3:**
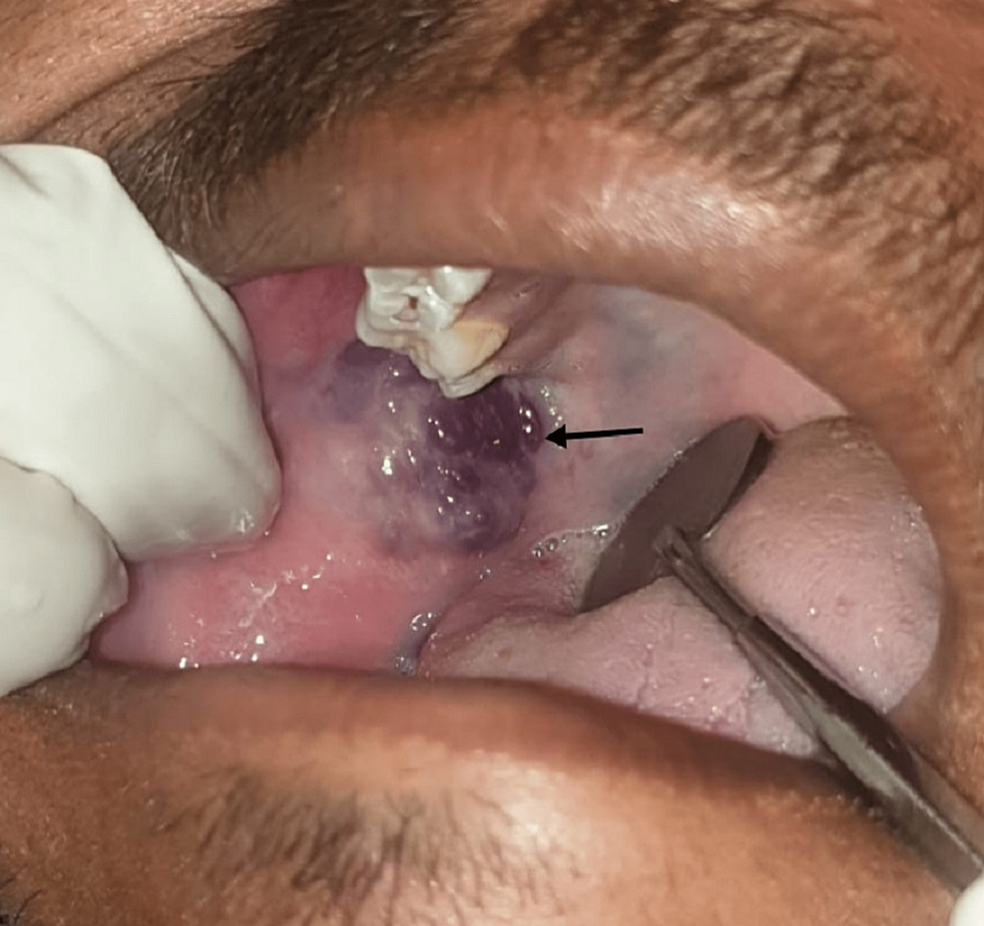
On manipulation, the lesion was exaggerated and an elevated red-purplish lesion appeared in the posterior part of the mucosa

Maxillo-mandibular bony morphology was within the standard limit in the vicinity of the swelling. On bimanual palpation, the lesion was soft and compressible, non-tender, and had a diffuse periphery with evident pulsation. While palpating, the lesion was exaggerated, and an elevated red-purplish lesion appeared in the posterior part of the mucosa (Figure 3).

**Figure 4:**
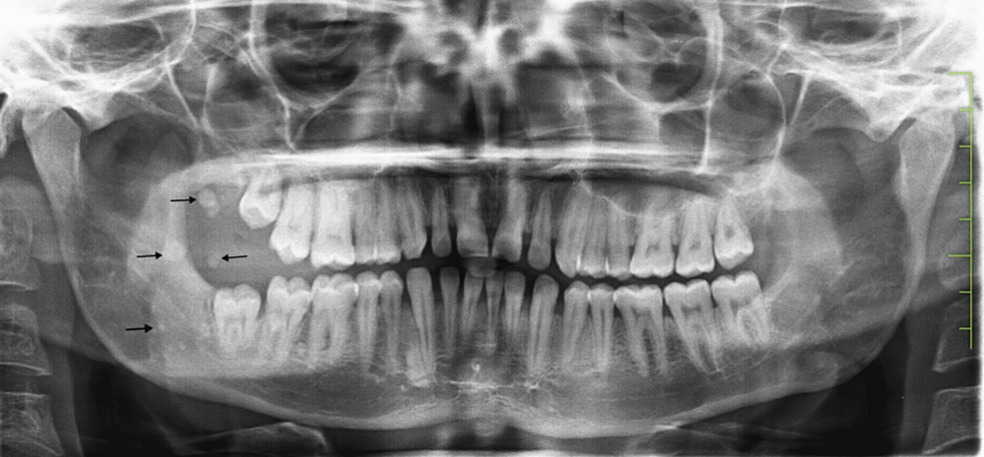
Panoramic image The image is showing multiple concentric circles suggestive of phleboliths in the right ramus region.

**Figure 5:**
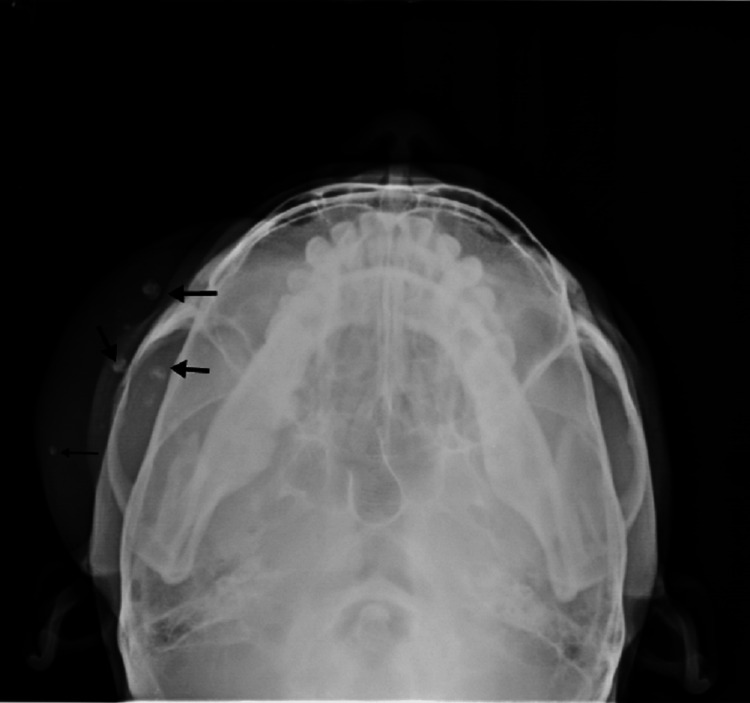
Submentovertex radiograph The radiograph image is showing multiple concentric calcifications in the soft tissue shadow of the lesion suggestive of phleboliths.

All the teeth were in good condition. Only extrinsic stains and calculus were there. Panoramic and submentovertex radiographs showed the presence of multiple concentric calcifications in the soft tissue shadow of the lesion suggestive of phleboliths (Figures 4, 5).

Assuring the benign nature of the disease and possible definite treatment, the patient was referred to the department of interventional radiology for management.

## Discussion

Scott reported a series of 393 IMHs in 1957; 12% were reported in the head and neck region [6]. The masseter muscle is the most frequent, accounting for 5% of all intramuscular hemangiomas. The trapezius, periorbital, sternocleidomastoid, and temporalis muscles follow the masseter muscle in frequency [7]. Etiological factors play a viable role, like excessive muscle contraction and trauma and hormonal variations. It shows apparent increased volume correlated to menarche, menstrual cycle, and pregnancy [1], although, for masseteric locations, there is a male preponderance [3]. Most hemangiomas are recognised clinically and do not require any investigation. The chief complaint is the presence of a slowly enlarging mass [7,8]. The extraoral swelling compromised the aesthetics, which was the primary concern of this case. Intramuscular hemangiomas rarely display clinical symptoms or signs that reveal their vascular nature. They usually present with normal overlying skin, although there may be occasional reddish-blue discolouration. Thrills, bruits, compressibility, and pulsation are generally absent; however, pain can be present [7]. These features were also consistent with the present case.

The Wattle sign that the size of the tumour increases by lowering the position of the head helps in diagnosis was positive in this case. Precautions should be taken while examining the facial region as new vascularised tissue is more torturous and fragile as compared to the native vessels, and this may lead to submucosal bleeding and may lead to subepithelial hematoma formation [9]. We experienced some while palpating the lesion intraorally. Phleboliths in conventional soft-tissue radiology can be distinguished from intra-parotid sialoliths [2,8]. In approximately 25% of intramuscular hemangioma, phlebolith formation is present [5,7]. The thrombi are produced due to slowing peripheral blood flow, leading to the formation of phleboliths, which are later organised and mineralised. The mechanism of phlebolith formation is supposed to be thrombus calcification, which forms the centre of the phlebolith. This explains the concentric histopathologic structure of the phlebolith on the cut surface. Then there is secondary calcification of the fibrinous component, which gets attached. The exact process continues, which causes an increase in the size of the phlebolith [5].

The vascularity of the mass can be confirmed by EchoDoppler, but cannot accurately decide contours and associations with surrounding tissue, which can be done with contrast-enhanced computed tomography and exclusively on magnetic resonance imaging (MRI): these investigations have become essential before the treatment [10]. Biopsy and fine needle aspiration are contraindicated as they can lead to severe haemorrhage. For large tumours, arteriography is indicated to identify the arterial pedicle, the main supply, and preoperative embolisation, hence decreasing the risk of preoperative bleeding [11]. Various non-surgical treatments are available, like sclerosant or corticosteroid injection, isolated embolisation, cryotherapy, arterial ligature, or radiation therapy. However, results have been debatable, and these methods are nowadays suggested only when surgery is contraindicated or not indicated [11-13]. Indications for complete surgical resection are large, aesthetically unpleasing tumour size, pain, and functional disability [13]. Preoperative embolisation is suggested for giant tumours. Numerous approaches are mentioned in the literature. Parotidectomy is the commonest as it gives sufficient exposure, although it needs dissection of the facial nerve and partial parotidectomy [14].

## Conclusions

Hemangioma of the face, in this case, was diagnosed based on the typical clinical presentation and using conventional radiography. The presence of small hard nodules that are compressible and diffuse should alert a clinician to the possible presence of an intramuscular hemangioma. X-ray images can show phleboliths and help diagnose them, whereas to identify the exact location of the calcification, non-ionizing techniques such as MRI and ultrasound can be helpful. The management is usually conservative, and surgery is the last treatment of choice.
